# Who will keep patients safe? The largest multi-centre survey of healthcare students exposes critical gaps in radiation safety education

**DOI:** 10.1186/s12909-026-09531-x

**Published:** 2026-05-26

**Authors:** Arkadiusz Szarmach, Monika Waśkow, Sebastian Glowinski, Paweł Gać, Rafał Poręba, Łukasz Rypicz, Maciej Piskunowicz, Marcel Zoch, Katarzyna M. Michalak, Edyta Szurowska, Alexander Ramanowski, Magdalena Wszędybył-Winklewska

**Affiliations:** 1 2nd Department of Radiology, Medical University of Gdansk, Gdansk, 80-210 Poland; 2 Institute of Health Sciences, Pomeranian University in Slupsk, Slupsk, 76-200 Poland; 3 Centre for Diagnostic Imaging, 4th Military Hospital, Weigla 5, Wroclaw, 50-981 Poland; 4 Department of Environmental Health, Occupational Medicine and Epidemiology, Wroclaw Medical University, Mikulicza-Radeckiego 7, Wroclaw, 50-368 Poland; 5 Department of Biological Principles of Physical Activity, Wroclaw University of Health and Sport Sciences, Paderewskiego 35, Wrocław, 51-612 Poland; 6 Department of Public Health, Wroclaw Medical University, Chałubińskiego 3, Wrocław, 50-368 Poland; 7 1st Department of Radiology, Medical University of Gdansk, Gdansk, 80-210 Poland; 8 Students’ Scientific Association, Faculty of Medicine Ludwik Rydygier Collegium Medicum, Nicolaus Copernicus University in Bydgoszcz, Bydgoszcz, 85-067 Poland; 9 Department of Neurophysiology, Neuropsychology and Neuroinformatics, Medical University of Gdansk, Gdansk, 80-210 Poland; 10 Department of Psychiatry and Psychotherapy, Helios Klinik, Hettstedt, Germany

**Keywords:** Medical education, Radiation safety, Radiological protection, Student knowledge, Ionising radiation

## Abstract

**Background:**

The increasing use of ionising radiation in medical diagnostics highlights the need for adequate education of future healthcare professionals in radiation protection. This study aimed to assess the level of radiation safety knowledge among healthcare students across different disciplines and years of study.

**Methods:**

A cross-sectional survey was conducted among 1,161 students from three Polish medical universities, representing medicine, nursing, radiography, physiotherapy, and paramedic programmes. The questionnaire evaluated knowledge of radiation protection principles, identification of imaging modalities involving ionising radiation, and estimation of relative radiation doses.

**Results:**

The mean percentage of correct answers was 30.1% (SD = 13.9%), indicating generally low levels of knowledge. Fifth-year students achieved the highest scores (41.7%), while fourth-year students performed the worst (23.4%). Physiotherapy students obtained the highest mean scores (36.7%), whereas paramedic students scored the lowest (25.9%). Significant differences were observed between years of study and fields of study (*p* < 0.001), although effect sizes were small. No significant differences were found between universities (*p* = 0.063). Students performed best in identifying imaging modalities and worst in radiation dose estimation. Notably, 21% of respondents reported no interest in radiation protection.

**Conclusions:**

Healthcare students demonstrate insufficient knowledge of radiation protection, with only limited improvement across years of study. These findings highlight the need for structured, curriculum-integrated education to improve awareness and safe use of ionising radiation.

**Supplementary Information:**

The online version contains supplementary material available at 10.1186/s12909-026-09531-x.

## Introduction

Modern medicine relies heavily on advanced diagnostic and therapeutic technologies, with ionising radiation playing a pivotal role in many clinical applications [1–3]. Currently, the principal medical uses of ionising radiation include diagnostic imaging, interventional radiology, nuclear medicine, and radiotherapy [4]. Consequently, exposure to ionising radiation has become an almost unavoidable occupational factor for healthcare professionals, irrespective of their specialty.

The global increase in procedures involving radiation is driven by various factors, including demographic shifts (e.g. population ageing), greater availability of high-end medical equipment, enhanced diagnostic accuracy, and rising patient expectations. While these technologies offer substantial clinical benefits, they also raise important concerns regarding patient and staff safety, particularly in relation to radiation protection.

Healthcare workers, regardless of their specific roles, should possess at least a basic understanding of the biological effects of ionising radiation, including both deterministic and stochastic effects. Deterministic effects occur above a threshold dose and are dose-dependent; clinical manifestations such as nausea, skin injury, cataracts, or gastrointestinal damage may appear shortly after exposure, with severity increasing alongside the dose. By contrast, stochastic effects—such as radiation-induced malignancies—are probabilistic in nature. They do not depend on dose magnitude but rather on cumulative exposure, with the risk increasing proportionally. Stochastic effects often manifest years after exposure and pose a particular risk to children due to their longer expected lifespan [5, 6].

Effective radiation protection is essential for the safe clinical application of ionising radiation. This requires that medical personnel are thoroughly educated in the principles of radiation safety and are fully aware of the potential consequences of improper radiation use. Inadequate practices can significantly elevate the risk of adverse health outcomes, including site-specific cancers [7–9]. It has been estimated that up to 2% of all malignancies may be attributable to radiation exposure [10], and the risk of stochastic effects is estimated to increase by approximately 5% per 1 Sv of effective dose [11, 12].

In light of this, education in radiation protection should be considered a core component of healthcare training. Numerous studies have assessed knowledge levels in this area among medical students, physicians, and other healthcare professionals [13–15]. However, most existing research has been limited to single- or dual-centre studies with relatively small sample sizes. In contrast, the present study surveyed over 1,100 students from various medical universities in Poland, encompassing different academic years and healthcare disciplines. To the best of our knowledge, this is the first large-scale, multi-centre assessment of its kind in this population.

The aim of this study was to conduct the largest and most comprehensive assessment to date of healthcare students’ knowledge concerning the effects of ionising radiation and the principles of radiation protection. Additionally, the study investigated attitudinal factors, such as students’ interest in radiation safety, in order to provide a more holistic understanding of current educational gaps. The findings may support evidence-based curriculum development aimed at aligning radiation safety training with the evolving demands of modern healthcare practice.

## Materials and methods

A prospective, cross-sectional, multi-centre, anonymous, and confidential questionnaire-based survey was conducted among medical students from three Polish medical universities: the Medical University of Gdansk (MUG), Wroclaw Medical University (WMU), and the Pomeranian University in Slupsk (PUS), between 1 May and 31 December 2024. The students included in the study had not received formal, dedicated training in radiation protection prior to the study. Participation in the study was voluntary, and all participants provided verbal informed consent prior to completing the questionnaire. Students who did not provide consent were excluded. Additionally, 28 partially completed questionnaires were rejected from the analysis. The study protocol was reviewed and approved by the Bioethics Committee for Scientific Research at the Medical University of Gdansk, Poland (approval number: KB/244/2024). The study was conducted in accordance with the principles of the Declaration of Helsinki. Ultimately, a total of 1,161 students were included in the final dataset (Table 1).

**Table 1:** Distribution of students by university

University	Number of Students	Percent [%]
MUG	515	44.4
WMU	499	43.0
PUS	147	12.7

The survey instrument consisted of three sections:

- Section I included demographic questions concerning the respondent’s field and year of study.
- Section II contained general questions related to radiological protection and awareness.
- Section III, presented in tabular form, comprised specific knowledge-based questions that evaluated students’ understanding of radiation dose levels associated with ionising radiation used in various medical procedures, expressed in chest X-ray equivalents. Internal consistency of the knowledge items (Section III) was assessed using Cronbach’s alpha coefficient to evaluate the reliability of the questionnaire. The analysis was performed using the *psych* package in R (version 4.3.2; R Core Team, Vienna, Austria), and returned a Cronbach’s α coefficient of 0.84, indicating good internal consistency of the knowledge items. For the purposes of domain-level analysis, the 13 items of Section III were grouped as follows: items Q1–Q8 assessed students’ ability to estimate relative radiation doses associated with specific diagnostic procedures; item Q9 and Q10 assessed identification of imaging modalities as ionising or non-ionising; and items Q11–Q13 assessed knowledge of radiation protection principles, radiosensitivity, and shielding measures. The questionnaire focused primarily on diagnostic imaging procedures and practical aspects of radiation protection relevant to clinical decision-making, rather than detailed legislative or regulatory frameworks. Radiation doses were expressed in relative terms (as equivalents of a standard chest X-ray) rather than absolute units (e.g., mGy or mSv), to facilitate interpretation and comparison among students with varying levels of knowledge.

The student survey and correct (expected) answers to the questions included in Section III are provided in Appendix 1. A total of 147 questionnaires were completed online (PUS), while 1014 paper-based questionnaires were collected at MUG and WMU. Students had a maximum of 10 min to complete the survey, regardless of the format (online or paper-based).

Outliers were identified using Grubbs’ test. The normality of data distribution was assessed using the Shapiro–Wilk, Lilliefors, Kolmogorov–Smirnov, and Jarque–Bera tests, and equality of variance between groups was tested using Levene’s test (Brown–Forsythe modification). To assess the significance of the differences between two independent groups, the Student’s t-test was used when the assumptions of normality and homogeneity of variance were met; otherwise, Welch’s t-test was applied. For non-normally distributed variables, the Mann–Whitney U test was used. Quantitative variables were described using the mean (x̄), standard deviation (SD), median (Me), minimum (Min), maximum (Max), and coefficient of variation (V). Qualitative variables were presented in tabular form as absolute values and percentages.

## Results

### Respondent demographics

Second-year students constituted the largest group among all academic years (*n* = 543). Questions 1 and 2 concerned the respondents’ year and field of study, respectively; Table 2 additionally presents the number and proportion of students within each subgroup who correctly identified their academic year and field of study. The highest rate of correct identification was observed among fifth-year students (48.1%), whereas second-year students showed the lowest rate (16.6%).

**Table 2:** Demographic characteristics of study participants (Questions 1 and 2)

Year of study	Number of students	Number of students with correct response (Q1-Q2)^*^	Percentage [%]
1st year	284	71	25.0
2nd year	543	90	16.6
3rd year	45	14	31.3
4th year	156	37	23.7
5th year	133	64	48.1
**Field of Study**			
Medical faculty	703	157	22.3
Nursing	229	40	17.5
Radiography	103	36	35.0
Physiotherapy	67	33	49.3
Paramedic	59	10	16.9

^*^The number of correct responses refers to the combined results of Questions 1 and 2

### General questions on radiation protection

When asked about the sources of their knowledge on radiation protection, most students indicated university classes as their primary source (71.4%), followed by media (28.4%). A smaller proportion reported that they had not sought information on this topic (21.1%), while medical journals were the least frequently cited source (6.2%) (Table 3). This indicates that formal education remains the dominant source of knowledge in this area. Percentages are calculated based on the total number of participants, and multiple responses were allowed; therefore, percentages do not sum to 100%.

**Table 3:** Where did you obtain your knowledge on radiation protection in medical procedures involving ionising radiation? (Question 3). (Multiple responses were allowed)

Source of knowledge	Number of responses	% of total participants
Lectures/seminars	829	71.4
Media	330	28.4
Medical journals	72	6.2
I don’t know	245	21.1
Other	28	2.4

The gonads (82.1%) and bone marrow (69.8%) were most frequently identified by respondents as organs particularly sensitive to ionising radiation, followed by the lens of the eye (52.9%). In contrast, only a small proportion of participants indicated skeletal muscles (2.6%) as radiosensitive organs. Regarding vulnerable groups, the majority of respondents correctly identified children as the most sensitive population to ionising radiation (70.2%). However, 26.2% of participants believed that age does not play a significant role in radiation risk, indicating gaps in knowledge in this area (Table 4).

**Table 4:** Knowledge of students about the groups and organs exposed to Ionizing Radiation (Question 4 and 5)

Age group	Number of students	Percentage [%]
Children	815	70.2
Age > 65 yo	40	3.5
Age does not matter	304	26.2
**Organs at Risk**		
Lens of the eye	614	52.9
Gonads	953	82.1
Bone marrow	810	69.8
Stomach	127	10.9
Kidneys	118	10.2
Muscles	30	2.6
None of above	7	0.6

A very high proportion of students (95.2%) correctly identified lead aprons as an effective method of protection against ionising radiation. Additionally, the majority of respondents recognised maintaining distance from the radiation source as a protective measure (87.8%). Notably, 18.2% of participants incorrectly considered a Faraday cage to be effective for radiation protection, indicating the presence of misconceptions in this area (Table 5).

**Table 5:** Which protective measures against ionising radiation are you familiar with? (Multiple responses allowed; responses do not sum to 100%) (Question 6)

Protection methods	Number of students	Percentage [%]
Lead apron	1105	95.2
Distance from the radiation source	1019	87.8
Dosimeter	300	25.8
Faraday cage	211	18.2
Diet rich in carbohydrates	17	1.5

### Detailed knowledge of radiation doses and procedures

In the final section of the questionnaire, respondents were asked to estimate radiation doses associated with selected diagnostic procedures. Rather than requiring exact dose values (in mSv), the questions were based on relative comparisons to the dose from a standard chest X-ray, which served as a reference point. Additionally, this section assessed whether students were able to distinguish between imaging procedures that involve ionising radiation and those that do not. The internal consistency of the knowledge items was good (Cronbach’s α = 0.84). The mean percentage of correct answers among all 1,161 students was 30.1% (SD = 13.9%), with a median of 23.7%. Question 7 had the lowest proportion of correct responses (16.6%), whereas Question 9 had the highest (69.1%) (Table 6).

**Table 6:** Statistics of correct and incorrect responses [%] for the total sample (*N* = 1,161 students)

Question	Correct (%)	Incorrect (%)
Q1 – abdominal X-ray dose	42.1	57.9
Q2 – lumbar spine X-ray dose	34.9	65.1
Q3 – barium enema dose	21.4	78.6
Q4 – peripheral angiography dose	21.5	78.5
Q5 – head CT dose	28.9	71.2
Q6 – chest CT dose	23.7	76.3
Q7 – abdominal CT dose	16.6	83.4
Q8 – Voiding Cystourethrogram dose (VCUG)	23.5	76.4
Q9 – abdominal ultrasound	69.1	30.9
Q10 – Doppler ultrasound	39.4	60.6
Q11 – thyroid nuclear scan	23.2	76.8
Q12 – brain MRI	25.8	74.2
Q13 – mammography	21.5	78.6

**Fig. 1:**
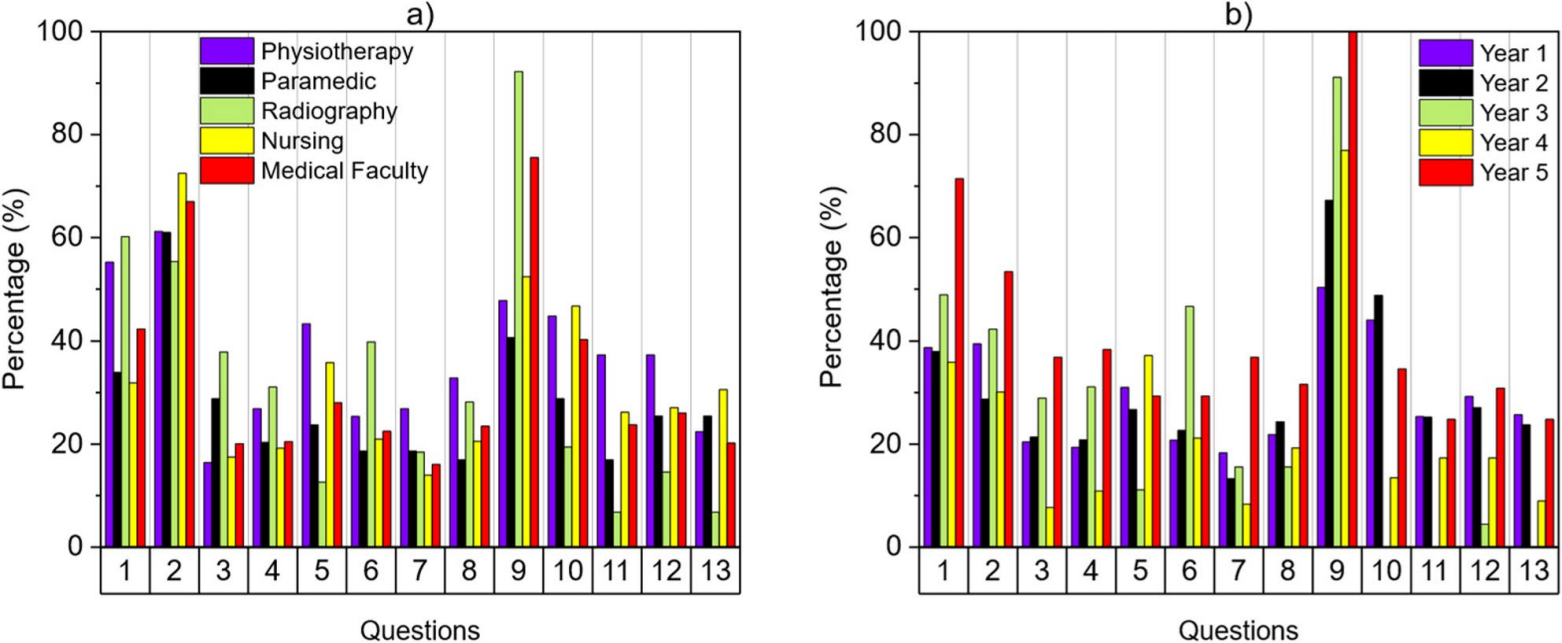
Percentages of correct responses by: (**a**) study faculty and (**b**) study year

Regardless of the faculty of study or the academic year, Question 9 (abdominal ultrasound) was the easiest, whereas Question 7 (CT of the abdomen and pelvis) proved to be the most difficult for all groups (Fig. 1a and b).

Statistically significant differences in knowledge scores were observed between years of study (Kruskal–Wallis, χ² = 25.73, *p* < 0.001) and fields of study (χ² = 24.85, *p* < 0.001). However, no statistically significant differences were found between universities (χ² = 5.53, *p* = 0.063). The effect sizes were small for both comparisons (years of study: eta-squared = 0.019; fields of study: eta-squared = 0.018), indicating that although statistically significant, these differences were of limited practical importance.

Post-hoc analysis (Dunn test with Bonferroni correction) showed that paramedic students achieved significantly lower scores than students of physiotherapy (*p* < 0.001), radiography (*p* < 0.001), and the medical faculty (*p* = 0.014). In addition, radiography students scored higher than nursing students (*p* = 0.046). No other pairwise comparisons remained significant after adjustment for multiple testing.

With respect to the year of study, fifth-year students achieved the highest scores, whereas lower years demonstrated poorer performance. The highest mean score was observed among fifth-year students (41.7%), while the lowest was recorded among fourth-year students (23.4%) (Table 7).

**Table 7:** Average percentage of correct answers by year of study, field of study, and university

University	Mean score (%) ± SD	Median	95% CI
MUG	29.8 ± 23.3	23.1	27.8–31.8
WMU	27.8 ± 20.7	23.1	26.0–29.6
PUS	39.1 ± 32.9	23.1	33.8–44.5
**Year of study**			
1st year	29.6 ± 25.2	23.1	26.6–32.5
2nd year	29.8 ± 21.8	23.1	28.0–31.7
3rd year	25.8 ± 10.5	23.1	22.7–28.9
4th year	23.4 ± 16.2	23.1	20.9–26.0
5th year	41.7 ± 34.4	23.1	35.9–47.5
**Field of study**			
Physiotherapy	36.7 ± 28.6	30.8	29.9–43.6
Paramedic	25.9 ± 27.1	15.4	19.0–32.9
Radiography	31.7 ± 19.7	30.8	27.9–35.6
Nursing	28.5 ± 23.2	23.1	25.5–31.5
Medical faculty	30.1 ± 23.9	23.1	28.4–31.9

Values are presented as mean ± SD (95% confidence interval). Group differences were analyzed using the Kruskal–Wallis test

Regarding the field of study, physiotherapy students achieved the highest overall knowledge scores (mean = 36.7%), whereas paramedic students obtained the lowest (mean = 25.9%). Differences between universities were less pronounced; students from PUS achieved the highest mean score (39.1%), while those from WMU had the lowest (27.8%) (Table 7). Despite differences in mean values, the median score remained identical across universities (23.1%), reflecting the discrete nature of the score distribution, with most students achieving 3–4 correct answers. When analysed by knowledge domains, students performed worst in radiation dose estimation (mean = 25.5%), whereas higher scores were observed for identification of imaging modalities (mean = 37.4%).

**Fig. 2:**
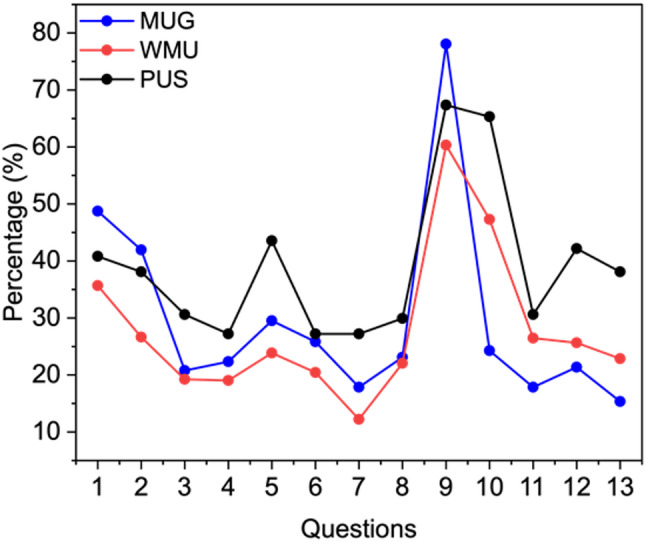
Percentages of correct responses to selected questions across universities

Across all universities, the question related to ultrasound was consistently answered most accurately, while the most challenging was again found to be the one concerning abdominal CT (Question 7) (Fig. 2).

**Fig. 3:**
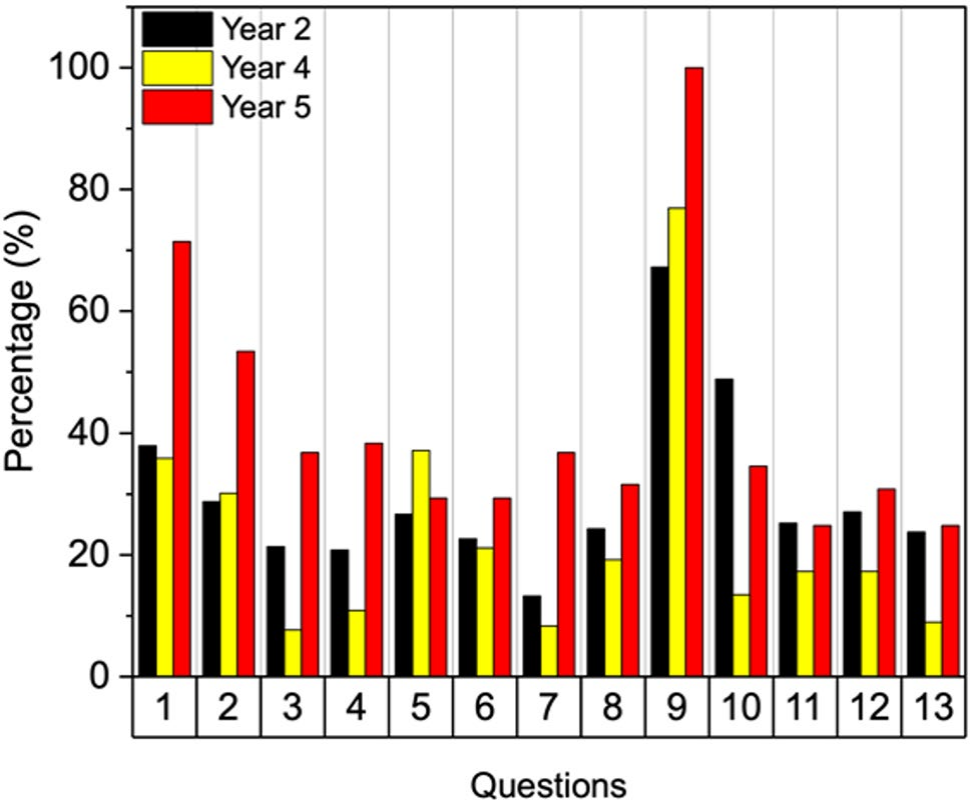
Percentages of correct responses by academic year: medical students

**Fig. 4:**
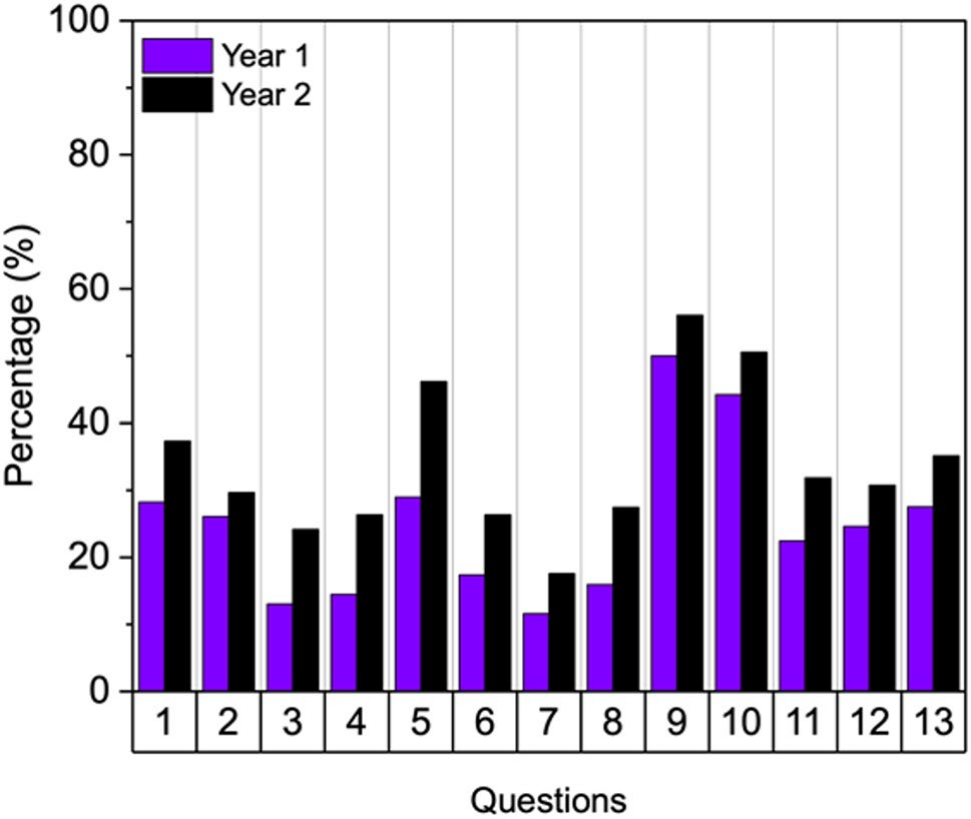
Percentages of correct responses by academic year: nursing students

An interesting learning curve was observed among medical faculty and nursing students, with a clear trend of increasing knowledge with each successive academic year (Figs. 3 and 4).

**Fig. 5:**
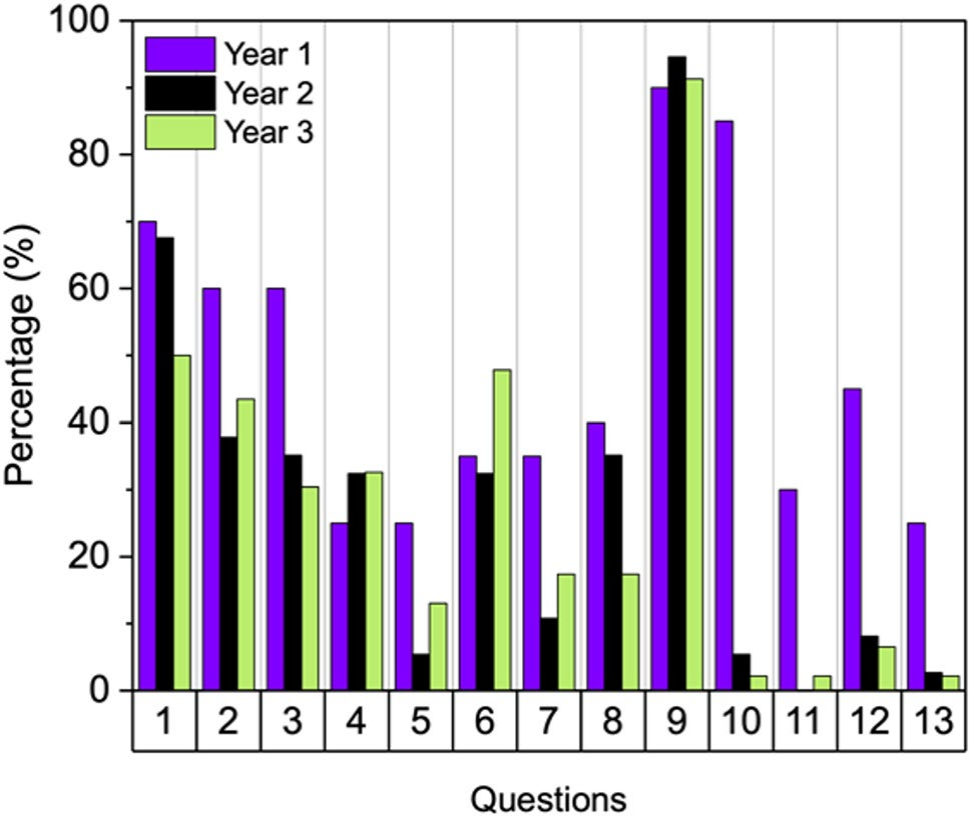
Percentage of correct responses by academic year: radiography students

However, no such pattern was observed among radiography students, with first-year students unexpectedly performing better than their more senior peers (Fig. 5).

## Discussion

Since the 1990s, the number of imaging examinations has increased substantially, resulting in a corresponding rise in patient exposure to ionising radiation [16, 17]. Among these, computed tomography (CT) is one of the most frequently performed diagnostic procedures and is associated with relatively high radiation doses compared with other imaging modalities [18–20]. According to the latest report by the United Nations Scientific Committee on the Effects of Atomic Radiation (UNSCEAR), the annual global number of X-ray-based procedures includes approximately 2.6 billion conventional radiographic examinations, 1.1 billion dental radiographs, 400 million CT scans, 24 million interventional procedures, and 40 million nuclear medicine studies [21].

A standard chest X-ray is associated with an effective dose of approximately 0.01–0.1 mSv, whereas chest CT typically ranges from 2 to 20 mSv. Even higher doses may occur in interventional radiology (5–70 mSv), while nuclear medicine procedures range from 0.3 to 70 mSv. For comparison, the average annual exposure to natural background radiation is approximately 3 mSv [22]. According to the BEIR VII report, exposure of ≥ 100 mSv is associated with an estimated cancer risk of approximately 510 cases per 100,000 individuals [23, 24].

Given the increasing use of radiation-based procedures, effective education in radiation protection is essential for future healthcare professionals. While knowledge of radiation doses is important, appropriate selection of imaging modalities remains a key component of clinical decision-making. These competencies should be regarded as complementary rather than competing, although the required depth of knowledge may vary depending on the future professional role.

To the best of our knowledge, this is the largest multi-centre study assessing radiation safety knowledge among healthcare students across different disciplines and academic years. The overall level of knowledge observed in our study was low, with a mean of 30.1% correct answers. This finding is consistent with previous reports indicating insufficient awareness of radiation-related risks among healthcare students and professionals [25–27].

Significant differences in knowledge scores were observed between years of study and fields of study; however, the effect sizes were small, indicating that knowledge gaps are widespread rather than confined to specific subgroups. Although fifth-year students achieved the highest scores, the overall level of knowledge remained insufficient even in this group. This suggests that progression through the curriculum alone does not ensure adequate acquisition of radiation safety knowledge [28–35].

Differences between fields of study may reflect variation in professional roles and curricular emphasis. Physiotherapy students achieved the highest scores, which may stem from a stronger emphasis in their curriculum on the practical aspects of diagnostic imaging, including patient safety, whereas paramedic students performed the worst. In Poland, paramedic training is primarily focused on emergency care and rapid patient management, with limited involvement in imaging referral decisions, which may partly explain the lower emphasis on radiation protection within their curriculum.

In the Polish healthcare system, the authority to request diagnostic imaging examinations is restricted to licensed physicians. Radiographers play a key role in implementing optimisation principles at the point of examination, while radiologists are responsible for ensuring the justification of imaging procedures in accordance with the ALARA (As Low as Reasonably Achievable) principle. Under EU Council Directive 2013/59/Euratom, justification of each exposure to ionising radiation is a legal requirement. Therefore, at least a basic level of radiation dose awareness is necessary for clinicians involved in patient management and referral decisions.

Analysis of knowledge sources indicated that most students relied on university teaching, whereas relatively few reported using scientific literature. Notably, approximately one-fifth of respondents declared no interest in radiation protection, which may have implications for future patient safety. This finding should, however, be interpreted with caution, as the question did not assess the timing or intensity of exposure to educational content. When this proportion was examined by field of study, it was highest among paramedic students, consistent with the limited relevance of imaging referral in their anticipated professional role, and lowest among radiography students, for whom radiation safety constitutes a core occupational competency. A more granular analysis of attitudinal and motivational factors across professional groups was beyond the scope of the present study and represents a direction for future research.

Encouragingly, a majority of students correctly identified children as the most radiosensitive group and recognised key radiosensitive organs such as the gonads, bone marrow, and lens of the eye. These findings are broadly consistent with previous studies, although some discrepancies across populations remain [27, 36, 37].

Knowledge of protective measures was relatively high, particularly regarding the use of lead aprons and maintaining distance from the radiation source. However, the incorrect identification of a Faraday cage as a protective method by a notable proportion of respondents indicates the presence of conceptual misunderstandings. 

Domain-based analysis revealed that students performed worst in radiation dose estimation, whereas higher scores were observed for identification of imaging modalities. This is in line with previous reports showing that students more readily recognise imaging modalities than accurately estimate radiation doses [30, 31]. Consistent with this observation, ultrasound was most frequently identified correctly, while CT-related questions showed the lowest accuracy.

Differences between universities were not statistically significant, suggesting a relatively uniform level of knowledge across institutions. These differences should nonetheless be interpreted with caution, given the small sample size from PUS (*n* = 147) and potential differences in curricular emphasis between institutions.

### Strengths

The strengths of this study include its large sample size, multi-centre design, and inclusion of students from different healthcare disciplines and stages of education, allowing for a comprehensive assessment of radiation knowledge.

### Limitations

This study has several limitations. First, the use of both paper-based and electronic questionnaires may have affected data consistency. Second, the study assessed declarative knowledge, which does not necessarily translate into practical competencies. Third, the sample was limited to three universities in Poland; therefore, the findings should be interpreted within the context of the Polish medical education system and may not be directly generalisable to other countries.

The questionnaire primarily focused on diagnostic imaging procedures, with limited inclusion of nuclear medicine and no representation of radiotherapy. In addition, detailed curricular characteristics, e.g., teaching hours, methods, or ECTS (European Credit Transfer and Accumulation System) were not analysed, limiting interpretation of inter-institutional differences. Radiation doses were expressed using relative categories rather than absolute units, which may limit direct comparison with standard dosimetric values. Moreover, the questionnaire did not include a dedicated item explicitly assessing knowledge of ionising versus non-ionising modalities, although this distinction was evaluated indirectly.

The unequal distribution of participants across academic years, particularly the overrepresentation of second-year students, may have influenced comparisons between groups. Furthermore, potential confounding factors such as age, gender, and prior clinical experience were not assessed. Future studies should incorporate these variables and include structured comparisons of curricula to better understand the determinants of radiation knowledge.

## Conclusions and future directions

As future healthcare professionals, students must develop a solid understanding of the safe use of ionising radiation in clinical practice. This includes familiarity with both ionising and non-ionising imaging modalities, associated exposure levels, and potential health risks for patients and healthcare workers. However, the required depth of knowledge is not uniform across disciplines: while detailed dose literacy is essential for physicians and radiographers, a broader understanding of radiation protection principles may be sufficient for other professional groups.

The present study demonstrated that, irrespective of university, year of study, or healthcare discipline, students possess only a basic level of knowledge regarding radiation exposure. Although knowledge levels tended to increase with advancing years of study, this improvement remained limited, and overall knowledge was insufficient even among senior students. Furthermore, despite statistically significant differences between years of study and fields of study, the small effect sizes indicate that knowledge gaps are widespread rather than confined to specific subgroups, suggesting a systemic rather than discipline-specific educational deficit.

These findings highlight the need for structured, curriculum-integrated approaches to radiation safety education. Future research should investigate in greater detail which curricular components — including teaching hours, instructional methods, simulation-based learning, and e-learning resources — are most effective in addressing these gaps. International comparative studies may help identify best practices that can be adapted across different healthcare education systems.

In light of these findings, and given the growing role of digital technologies in medical education, the development of an AI-powered mobile application available to students from the early stages of training warrants consideration. Such a tool could assist users in estimating radiation doses associated with common imaging procedures, analogous to clinical decision-support calculators used in patient management. It could also support clinical decision-making by guiding users toward the most appropriate imaging modality based on clinical presentation, thereby reinforcing both dose awareness and evidence-based referral practices.

This approach aligns with the recommendations of the EURAMED rocc-n-roll initiative, which advocates the integration of modern digital tools into radiation safety education across Europe [38]. Moreover, systematic reviews indicate that mobile learning applications can improve knowledge retention and learner engagement in medical education [39]. The integration of such tools into healthcare curricula may enhance not only factual knowledge but also professional attitudes toward the safe and justified use of ionising radiation, ultimately contributing to improved patient and occupational safety.

## Supplementary Information

Supplementary Material 1. Appendix 1.docx- A Survey on Students’ Knowledge of Radiation Protection.

## Data Availability

The datasets used and analyzed during the current study are available from the corresponding author on reasonable request.

## Abbreviations

ALARA: As Low as Reasonably Achievable
ECTS: European Credit Transfer and Accumulation System
CT: Computed Tomography
MUG: Medical University of Gdansk
WMU: Wroclaw Medical University
PUS: Pomeranian University in Slupsk
Sv: Sievert
mSv: millisievert
SD: Standard deviation
UNSCEAR: United Nations Scientific Committee on the Effects of Atomic Radiation
BEIR: Biologic Effects of Ionizing Radiation
US: Ultrasound
MR: Magnetic Resonance Imaging
VCUG: Voiding Cystourethrogram
