# Supplementary material for: Who will keep patients safe? The largest multi-centre survey of healthcare students exposes critical gaps in radiation safety education
Source: BMC Med Educ. 2026 May 26;26:1182. doi: 10.1186/s12909-026-09531-x (PMC13386553; doi:10.1186/s12909-026-09531-x)
Supplement: Supplementary file 1 — Supplementary Material 1. Appendix 1.docx- A Survey on Students’ Knowledge of Radiation Protection. [file 12909_2026_9531_MOESM1_ESM.pdf]

A Survey on Students' Knowledge of Radiation Protection

1. I am a student of:

a/ 1st year

b/ 2nd year

b/ 3rd year

c/ 4th year

d/ 5th year

2. I am studying at:

a/ medical faculty

b/ physiotherapy

c/ nursing

d/ radiography

e/ paramedic

3. Where did you obtain knowledge about radiation protection during medical procedures using ionizing radiation?

a/ during classes at the university

b/ from scientific journals

c/ from public media

d/ I was not interested in this topic

4. Who do you think is particularly sensitive to the adverse effects of ionizing radiation?

a/ children

b/ adults

c/ people over 65

d/ age doesn't matter

5. Organs that are particularly sensitive to radioactivity are: (multiple choices possible):

a/ eye lens

b/ kidneys

c/ gonads

d/ bone marrow

e/ muscles

f/ stomach

5. Methods of protection against ionizing radiation: (multiple selection possible):

a/ lead aprons

b/ distance from radiation source

c/ carbohydrate-rich diet

d/ dosimeters

e/ Faraday cage

6. ***What is the absorbed dose (per single chest X-ray) that a patient receives during the following types of imaging examinations? Mark the correct answer with X***

**No.**	**Type of imaging exam**	**0**	**1-9**	**10-49**	**50-99**	**100-199**	**200-299**	**300-399**	**400-499**
0	Chest X-ray		***X***						
1	Abdominal X-ray			***X***					
2	Lumbar spine x-ray			***X***					
3	Double contrast barium enema				***X***				
4	Limb arteriography					***X***			
5	Head CT exam without and after contrast			***X***					
6	Chest CT exam with and without contrast					***X***			
7	Abdominal CT exam with and without contrast							***X***	
8	Voiding cystourethrography (VCUG)				***X***				
9	Abdominal ultrasound exam	***X***							
10	Lower limb doppler exam	***X***							
11	Thyroid scintigraphy			***X***					
12	Brain MRI with and without contrast	***X***							
13	Mammography			***X***					
